# Advances in High-Performance Non-Ferrous Materials

**DOI:** 10.3390/ma16031186

**Published:** 2023-01-30

**Authors:** Hailiang Yu, Zhilin Liu, Xiaohui Cui

**Affiliations:** 1 Light Alloy Research Institute, College of Mechanical and Electrical Engineering, Central South University, Changsha 410083, China; 2 State Key Laboratory of High Performance Complex Manufacturing, Central South University, Changsha 410083, China

Non-ferrous metallic materials are considered to be fundamental materials for manufacturing in-dustries, i.e., they are used in aerospace, marine, vehicle, communications, construction, and even microelectromechanical systems. In order to obtain high-quality non-ferrous materials and/or components, we must fully understand the underlying process–structure–property relationship in the metallic materials such as Al alloys, Mg alloys, Ti alloys, Cu alloys, and etc. This Special Issue (SI), “*Advances in High-Performance Non-ferrous Materials*”, collected the latest development in fab-ricating the high-performance non-ferrous materials. Specifically, the coupled effects of alloying, microstructures, and processing on the mechanical properties of the representative non-ferrous alloys and related composites were elucidated in this SI. A few new processing technologies were also introduced that are superior in terms of the material strengthening, fracture toughness, me-chanical properties, magnetic properties, and wear resistance. Many of these efforts depend on the application of different new technologies such as irradiation, cryogenic rolling, wet chemical techniques, induction sintering, liquid/solid casting, heat treatments, electromagnetic hot forming, and five-axis flank milling.

Yang et al. [1] investigated both the tensile behavior and failure mechanism of the neutron-irradiated LT21 Al alloy. The significant variation of the tensile properties was due to the neutron transmutation-produced precipitates (i.e., Si and Mg_2_Si). Using electron backscatter diffraction, transmission electron microscopy, and X-ray diffraction, Li et al. [2] explored the effects of room temperature rolling and cryogenic rolling on the microstructure, textures, and mechanical properties of the Cu–Ti–Cr–Mg alloy. Material strengthening and microstructural evolution are closely related to the dynamic recovery and dynamic recrystallization that were impeded by cryogenic temperature. Meanwhile, the cryogenic temperature facilitates the production of dislocations and nano-twins, further enhancing the corresponding mechanical properties. Zhang et al. [3] studied the influence of cryogenic treatments on the microstructure and mechanical characteristics of Al–SiC composites. The cryogenic treatment increased the dislocation density and micro defects near the boundaries. More nucleation sites for precipitation during aging could be provided to increase the hardness and yield strength. Wang et al. [4] unraveled the microstructure–mechanical properties relationship of the W–Al_2_O_3_ composite that was prepared *via* induction sintering and rolling processes. Shuai et al.’s work revealed the alloying elements’ (i.e., La and Y) effects on the microstructure and mechanical properties of cast Al–Si–Cu alloys [5]. The addition of La and Y had an evident effect on refining and/or modifying α-Al grains, the eutectic Si phase, and β-Al_5_FeSi intermetallics. Huang et al. [6] elucidated the precipitation behavior of the T_B_(Al_7_Cu_4_Li) and S(Al_2_CuMg) phases of the 2195 Al–Cu–Li alloy at different homogenization temperatures.

Mao et al. [7] and Gao et al. [8] studied the bonding strength of Al-matrix laminated composites subjected to liquid/solid casting and roll bonding, respectively. Zhao et al. [9] analyzed the mechanical parameters of asymmetrical rolling. Ma et al. [10] investigated the influence of CeO_2_ particle size on the microstructure, synthesis mechanism, and refining performance of an Al–Ti–C Alloy. Yu et al. [11] explored the correlation between the grain refiner and fracture toughness in a 7050 Al alloy ingot and plate. Moreover, the tensile deformation and fracture behavior of a Ti–5Al–5Mo–5V–1Cr–1Fe alloy were studied in situ by Pan et al. [12]. The effect of the element cobalt on the magnetic properties of Ni_50-x_Co_x_Mn_35.5_In_14.5_ annealed ribbons was studied by Dubiel et al. [13]. Du et al. [14] investigated the deformation behavior and properties of a 7075 Al alloy under electromagnetic hot forming. Xu et al. [15] proposed an efficient approach to the five-axis flank milling of 7075 Al alloy spiral bevel gears. Zhang et al. [16] performed a correlation analysis of the microstructure and tribological properties of an *in situ* VC_p_ reinforced iron-based composite. Wang et al. [17] conducted systematic experimental work regarding the high temperature oxidation behavior of an equimolar Cr–Mn–Fe–Co high-entropy alloy. Li et al. [18] revealed the microstructural evolution of lamellar-structured high-purity Ni through cold rolling and cryo-rolling. Finally, Manu et al. [19] reviewed the roles of titanium in cast Cu alloys. Their review work summarizes various casting techniques to fabricate bronze alloys, mainly focusing on the microstructures, tensile properties, and tribological characteristics of Cu–Sn and Cu–Sn–Ti alloys [19]. All these theoretical and experimental works aforementioned in this SI will shed new light on fabricating the non-ferrous materials with high performances.
